# Routine Imaging Surveillance After Frontline ABVD Improves Outcome in High-Risk Hodgkin Lymphoma

**DOI:** 10.3390/cancers17193242

**Published:** 2025-10-06

**Authors:** Novella Pugliese, Marco Picardi, Annamaria Vincenzi, Claudia Giordano, Anna Lucania, Alessandro Severino, Claudia Salvatore, Massimo Mascolo, Paola Della Cioppa, Fabrizio Pane

**Affiliations:** 1 Department of Clinical Medicine and Surgery, Hematology Section, University of Naples “Federico II”, 80131 Naples, Italy or novypugliese@yahoo.it (N.P.); marco.picardi@unina.it (M.P.); claudia.giordano@unina.it (C.G.); alessandro.severino@unina.it (A.S.); fabrizio.pane@unina.it (F.P.); 2 Hematology Unit, Azienda Sanitaria Locale Napoli 1 Centro, 80147 Naples, Italy; anna.lucania@aslnapoli1centro.it (A.L.); paola.dellacioppa@aslnapoli1centro.it (P.D.C.); 3 Department of Public Health, University of Naples “Federico II”, 80131 Naples, Italy; claudia.salvatore@unina.it; 4 Department of Advanced Biomedical Sciences, Pathology Section, University of Naples “Federico II”, 80131 Naples, Italy; massimo.mascolo@unina.it

**Keywords:** Hodgkin lymphoma, follow-up, first relapse, post-recurrence survival

## Abstract

**Simple Summary:**

Although many people with advanced Hodgkin lymphoma respond well to initial treatment, about one-third eventually experience a return of the disease. Often, these relapses are not immediately noticeable and are only detected through routine imaging scans. However, there is ongoing debate about whether regular imaging after treatment is truly helpful. In this study, we compared two groups of patients: one monitored with scheduled imaging scans and another followed with standard clinical check-ups. We found that patients whose relapses were detected early by imaging had less aggressive disease at the time of recurrence and responded better to second-line treatment, leading to longer periods without further disease progression. These results suggest that regular imaging may help identify relapses at an earlier and more treatable stage. This could lead to changes in how high-risk Hodgkin lymphoma patients are monitored after their first treatment, potentially improving long-term outcomes.

**Abstract:**

**Background/Objectives**: Despite the high complete response (CR) rate to first-line therapy, approximately one-third of patients with advanced-stage Hodgkin lymphoma (HL) eventually relapse. In up to 30–50% of cases, relapses are subclinical, i.e., initially detected only by imaging procedures. However, there is no definitive consensus on the optimal surveillance strategy for high-risk HL patients. **Methods**: The purpose of this cohort study is to evaluate the long-term outcome of stage II-B/IV HL patients who relapsed under routine imaging surveillance (imaging cohort) compared to those who relapsed under conventional clinical monitoring (standard cohort). Follow-up in the imaging cohort systematically included FDG-PET/CT, ultrasonography, and/or chest X-ray. At relapse, patients were treated with the same approach (salvage therapy and autologous hematopoietic stem cell transplantation [AHSCT]) in both cohorts. **Results**: A total of 123 high-risk HL patients were assessed at their first relapse: 80 in the imaging cohort and 43 in the standard cohort. The 2-year event-free survival (EFS) was significantly higher in the imaging cohort compared to the standard cohort (70% vs. 37.2%, respectively; *p* = 0.001). Similarly, the CR rate following salvage treatment was greater in the imaging cohort as compared to the standard cohort (68.8% vs. 41.9%, respectively; *p* < 0.004). These differences were due to the capability of routine imaging surveillance to detect disease with more limited extension (early onset of clinically silent relapses) as compared to standard clinical monitoring, which was associated with relapsed disease in a more advanced stage. **Conclusions**: Our findings suggest that routine imaging surveillance in patients with high-risk HL leads to improved EFS detecting relapses, which were characterized by more favorable prognostic factors (low tumor burden), thus enabling the timely administration of salvage therapy.

## 1. Introduction

Hodgkin lymphoma (HL) is a highly curable disease. Indeed, according to the National Cancer Institute’s Surveillance, Epidemiology, and End Results database, the 5-year overall survival is approximately 85%, reaching a rate of more than 92% in patients with localized disease [1]. For patients who achieve remission after induction, there is currently a lack of consensus regarding the optimal method and frequency of surveillance. The routine use of imaging procedures for surveillance of patients with HL in complete remission (CR) is commonly performed both in clinical practice and in clinical trials and is suggested for the first two years after therapy completion [2] in some treatment guidelines. Indeed, surveillance imaging offers the theoretical benefit of detecting clinically silent relapses, which may allow for a prompt start of second-line therapy, leading to improved outcomes. Some groups have recently reviewed the appropriateness of routine surveillance in HL in clinical practice and recommended against routine surveillance imaging in this disease due to several issues [3,4,5,6,7,8]. First, the majority of patients with HL are cured with current treatment approaches and relapsed patients are still eligible for curative therapies, including novel agents and/or conventional high-dose chemotherapy with autologous hematopoietic stem-cell transplant (AHSCT). In addition, a clear indication for the optimal technique as surveillance imaging for HL patients is not yet established and some of the procedures may result in a high rate of false-positive scans. Importantly, the benefit in terms of clinical outcome for patients undergoing routine surveillance imaging is unclear and, therefore, does not seem to counterbalance the potential risk of cumulative radiation exposure and consequent slightly increased cancer incidence reported in cases undergoing follow-up by using radiological imaging after induction therapy [9].

Here, we describe a high-risk HL population study that compares the long-term outcome of a cohort of patients who relapsed under routine imaging surveillance to that of a cohort of patients who relapsed under standard clinical surveillance. The aim was to address the uncertainty about the role of systematically performed imaging surveillance in advanced-stage HL patients in remission after first-line therapy.

## 2. Materials and Methods

### 2.1. Patients and Treatments

This retrospective study begins from a series of 456 consecutive patients with high-risk HL treated at two hematology centers in Naples (Italy), Federico II University and Santa Maria di Loreto Mare (SMLM) Hospital. Patients were diagnosed between June 2001 and December 2009.

First-line treatment consisted of six cycles of adriamycin, bleomycin, vinblastine, and dacarbazine (ABVD) [3], followed by irradiation of bulky site (32 Gy) if needed [10,11], for all the patients in the series. Relapsed patients received a salvage therapy consisting of either DHAP [12] or IGEV [13] regimens, repeated for two or three cycles, along with hematopoietic stem cell collection for transplantation. Peripheral blood stem cells were collected via apheresis after mobilization with granulocyte colony-stimulating factor (G-CSF), administered after the first or second cycle of salvage chemotherapy. Patients who achieved at least partial response (PR) following salvage chemotherapy underwent AHSCT, using either FEAM [14] or TEAM [15] as conditioning regimens.

Following two cycles of salvage therapy, all initially involved disease sites were re-evaluated by using appropriate modalities. Post AHSCT, treatment response was assessed using a combination of clinical evaluation, laboratory testing, and imaging techniques, including fluorodeoxyglucose (FDG) positron emission tomography (PET)/computed tomography (CT) scans [16,17]. Patients were subsequently monitored for a minimum of two years.

Regarding the definition of response, prior to 2007, lesions demonstrating 18-FDG uptake greater than the mediastinal background were classified as malignant [16]. Starting in 2007, patient responses were evaluated based on the criteria proposed by Cheson et al., whereby a negative PET scan was indicative of CR and a positive PET scan, in conjunction with a reduction of more than 50% in measurable disease, was consistent with PR [17].

This study was designed and conducted in accordance with the Declaration of Helsinki (October 2008, 59th World Medical Association General Assembly, Seoul, Korea). Ethical approval was obtained from all relevant committees, and all patients provided written informed consent. This study was registered at ClinicalTrials.gov database (ClinicalTrials.gov identifier, NCT04298619).

### 2.2. Inclusion and Exclusion Criteria

Inclusion criteria were (a) age 18–70 years; (b) histologically confirmed HL [18,19]; (c) Ann Arbor or Cotswold stage IIB with bulky disease and/or extranodal involvement or stage III–IV [20]; (d) achievement of CR confirmed by FDG PET/CT scans following completion of first-line therapy [16,17]; (e) histologically confirmed relapse during follow-up; and (f) eligibility for salvage therapy and AHSCT. Patients with comorbidities that could impair participation in follow-up procedures (i.e., obesity and diabetes mellitus, which are potential causes of uninterpretable ultrasonography [US] and FDG-PET imaging findings, respectively) were excluded from the imaging-based follow-up cohort. In contrast, such exclusions were not necessary in the standard surveillance cohort, where patients were followed clinically without reliance on advanced imaging modalities.

### 2.3. Follow-Up Procedures and Diagnosis of Relapse

The two centers applied different follow-up procedures after first remission. Patients followed at the University of Naples Federico II systematically underwent imaging-based surveillance (imaging cohort), while those followed at SMLM received the standard clinical surveillance (standard cohort). Long-term outcomes were compared between the two cohorts after relapse.

Post-remission surveillance in the imaging cohort consisted of systematic imaging assessments using either PET/CT or US scans combined with chest radiography (US/C-XR) for the evaluation of superficial, mediastinal, abdominal, and pelvic lymph nodes as integrated parts of an imaging-based follow-up protocol previously described in detail and further reported in the Supplementary Methods, together with a timeline figure illustrating the scheduled follow-up visits (Figure S1) [9].

Patients undergoing PET/CT and those receiving US/C-XR were pooled into a single imaging cohort (Figure S2), as prior results from a randomized trial demonstrated equivalent sensitivity of the two approaches in detecting both symptomatic and asymptomatic relapses [9].

In contrast, clinical surveillance for patients in the standard cohort (Figure S2) included medical history, symptom evaluation, physical examination, and routine laboratory testing (i.e., complete blood count, erythrocyte sedimentation rate [ESR], and serum chemistry). Imaging studies in the standard cohort were performed exclusively in cases where clinical suspicion of relapse arose [21].

All patients in both cohorts began post-remission follow-up in the fourth month following confirmation of CR and continued until occurrence of relapse or the end of the ninth year. Scheduled follow-up visits were conducted at fixed intervals, 4, 8, 12, 16, 20, 24, 30, 36, 48, 60, 84, and 108 months post-treatment completion, across both cohorts (Figure S1).

Suspected relapses by diagnostic work-up in both cohorts were confirmed histologically through lymph node biopsy, as already described [9]. At the time of relapse, comprehensive clinical and diagnostic data, including symptom profiles, physical findings, laboratory results, and restaging imaging, were systematically collected and recorded.

### 2.4. Study Endpoints

The primary objective of this study was to compare post-recurrence event-free survival (EFS) between the two patient cohorts. Post-recurrence EFS, hereafter referred to as second EFS, was defined as the time from the first disease relapse following induction therapy to the occurrence of an event or the date of last follow-up after AHSCT. Events were defined as (*i*) stable or progressive disease during salvage therapy, (*ii*) second relapse, or (*iii*) death from any cause. Second EFS was chosen as the primary endpoint because it reflects early treatment response in the post-relapse setting, which is critical for guiding subsequent clinical decisions such as proceeding to autologous stem cell transplantation (AHSCT), and it captures clinically meaningful events that directly impact patient management [22,23].

To assess potential lead-time bias related to early relapse detection [24,25], we also calculated time to first recurrence, hereafter referred to as first EFS. This was defined as the interval from the date of CR after first-line therapy to the date of first documented relapse. A comparative analysis between first EFS and second EFS was conducted to evaluate whether earlier detection of relapse translated into improved post-relapse outcomes.

Secondary endpoints included:

1. Overall EFS, defined as the time from CR following initial therapy to the occurrence of any event (as defined for second EFS) or the last follow-up. This measure was used to evaluate the long-term impact of follow-up strategies on disease control and second relapse-free survival.
2. First and second EFS among patients with asymptomatic (clinically silent) relapses, both in the overall study population and within the imaging cohort.
3. Response rates to salvage therapy, as assessed by FDG-PET/CT, both prior to and after AHSCT in each cohort.
4. The prognostic impact of established HL risk factors on EFS outcomes.

### 2.5. Statistical Analysis

All statistical analyses were performed using R software version 3.6.0. Patient characteristics between the active surveillance group and the treatment group were compared using Fisher’s exact test for categorical variables and the Mann–Whitney U test for continuous variables. Median follow-up time was estimated using the reverse Kaplan–Meier method.

Survival analyses were conducted using Kaplan–Meier curves, and differences between groups were assessed with the log-rank test. The proportional hazards (PH) assumption for Cox regression models was evaluated using Schoenfeld residuals and tested with the cox.zph function from the survival package. When the PH assumption was violated, Peto and Peto’s weighted log-rank test was applied to give greater weight to early differences in hazards.

Univariable Cox proportional hazards regression analyses were performed, and variables with *p*-values less than 0.01 were included in multivariable Cox regression models. Statistical significance was defined as a two-sided *p*-value less than 0.05.

#### Propensity-Score-Matched (PSM) Sub-Analysis to Mitigate Baseline Prognostic Differences

To further enhance the robustness of our findings and address potential baseline confounding not fully accounted for in the primary analysis, we performed an additional PSM sub-analysis. This supplementary approach was designed to improve covariate balance between the follow-up strategy groups and provide a more precise estimation of the effect of follow-up strategy on survival outcomes.

To adjust for potential baseline confounding, 1:1 PSM was performed without replacement using the MatchIt package in R, with optimal matching (method = “optimal”). The propensity score was estimated using a multivariable logistic regression model that included age at diagnosis, histological type, stage at diagnosis, and IPS score as covariates.

Covariate balance was assessed using standardized mean differences (SMD), with values < 0.10 considered acceptable. Matched pairs were identified via the subclass variable and used to define a pair_id for clustering.

Survival analysis was conducted using a Cox PH model (coxph function, survival package), incorporating cluster-robust standard errors to account for within-pair correlation. The PH assumption was tested and found to be satisfied.

## 3. Results

### 3.1. Relapse and Surveillance

After remission, patients were followed with either systematic imaging-based surveillance (*n* = 300) or classical clinical surveillance (*n* = 156). Details on patient characteristics, selection, exclusions, and group allocation are summarized in Supplemental data.

Overall, 123 consecutive patients with advanced or early-unfavorable HL who relapsed after achieving first CR were included in the final analysis. The characteristics at the time of initial HL diagnosis are reported in Table 1.

After a median follow-up of 33 months (range 4–108 months), relapses occurred in 43 patients (27.5%) in the standard cohort and 80 patients (26.6%) in the imaging cohort (Figure S2). Among the imaging cohort relapses, 29 were clinically overt, while the remaining 51 were detected solely by imaging without any clinical symptoms, accounting for 41.5% of all relapses in the study population. In contrast, in the standard cohort, all relapses were identified through clinically meaningful signs during follow-up visits and then confirmed by imaging tools and histology. Patient characteristics at relapse are summarized in Table 2.

**Figure 1:**
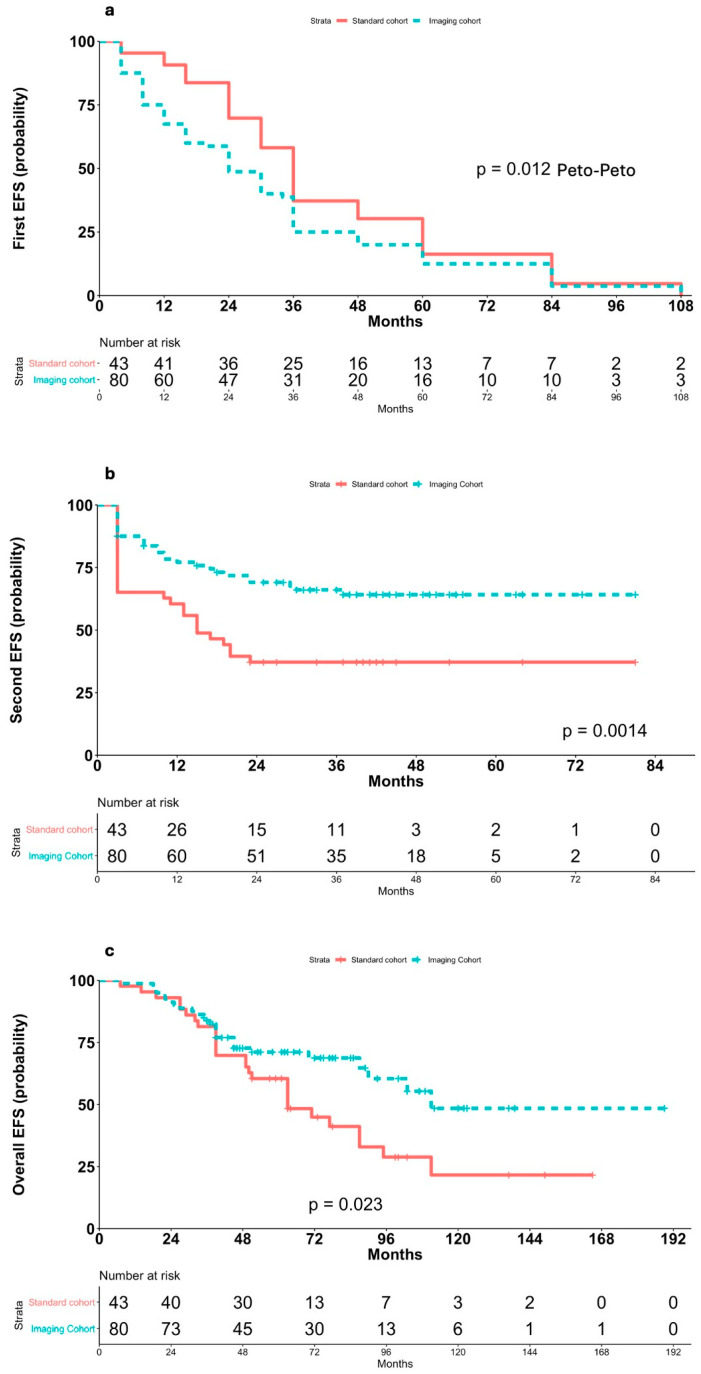
Kaplan–Meier analyses of EFS according to method of diagnosis recurrence. (**a**) First EFS in relapsed patients achieving standard and imaging-based follow-up after first complete remission according to Peto-Peto weighted log-rank test; (**b**) second EFS in relapsed patients achieving standard and imaging-based follow-up after first complete remission; (**c**) overall EFS in relapsed patients achieving standard and imaging-based follow-up after first complete remission.

The median time to relapse was 24 months [95% CI: 20–36] in the 80 patients of the imaging cohort, compared to 36 months [95% CI: 30–48] in the 43 patients of the standard cohort. The Schoenfeld residuals plot demonstrated a non-random pattern over time when comparing relapse timing between the two cohorts, indicating a violation of the proportional hazards (PH) assumption (*p* = 0.029). Therefore, the Peto-Peto weighted log-rank test, which gives greater weight to differences in hazards occurring earlier during follow-up and is more sensitive than the standard log-rank test when the relative risk between groups changes over time, was applied to the first EFS analysis. This test demonstrated that relapses were detected earlier in the imaging cohort (*p* = 0.012; Figure 1a). This earlier detection of relapse corresponded with differences in clinical features at recurrence between the two cohorts, specifically regarding disease stage and the proportion of bulky disease at relapse (Table 2).

### 3.2. Clinical Features at Relapse

At reassessment, approximately two-thirds of patients in the standard cohort relapsed at stage ≥ IIB, whereas only 42.5% of relapses in the imaging cohort were at stage ≥ IIB. This difference is largely attributable to the high proportion of clinically silent relapses in the imaging cohort (51 out of 80), which were typically diagnosed at an earlier stage (≤IIA) in 37 cases (72.5%) (Table 2). Furthermore, the proportion of patients presenting with bulky disease at relapse was significantly higher in the standard cohort compared to the imaging cohort (58.1% vs. 23.8%, *p* = 0.0003). Similarly, extranodal involvement was more common in the standard cohort than in the imaging cohort (20.9% vs. 8.8%, *p* < 0.05). These differences are mainly due to the subclinical detection of relapses in the imaging cohort (Table 2). Overall, clinically silent relapses in the imaging cohort were usually diagnosed at less advanced disease stages, without specific signs or symptoms of recurrence (i.e., no reported symptoms, abnormal blood tests, or physical examination findings), and were often located in deep anatomical sites (Table 2). Notably, no differences in outcome parameters were observed between patients monitored with PET/CT versus those monitored with US/C-XR.

### 3.3. Salvage Therapy and Response

Salvage therapy consisted of two or three cycles of either the DHAP or IGEV regimen, after which responses were reassessed by imaging and, when appropriate, biopsy.

Salvage regimens were comparable between cohorts; IGEV was administered to 76.7% (33/43) of patients in the standard cohort and 72.5% (58/80) in the imaging cohort, while DHAP was used in 23.3% (10/43) and 27.5% (22/80) of cases, respectively. There was no statistically significant difference in the distribution of salvage regimens between the two cohorts (*p* = 0.609), indicating that the type of second-line therapy received was comparable across groups.

A clinically meaningful response (CR plus PR) was observed in 28 of 43 patients (65%) in the standard cohort and in 68 of 80 patients (85%) in the imaging cohort (*p* < 0.01; Figure S2 and Table 3). Notably, a greater difference in the quality of responses was observed between the cohorts, with CR rates of 69% (55 patients) in the imaging cohort versus 42% (18 patients) in the standard cohort (Table 3).

The highest CR rate (84%) was observed in patients of the imaging cohort with clinically silent relapses, who overall exhibited limited disease spread at recurrence detection (72.5% of relapses at stage ≤ IIA; Table 1). The 68 patients in the imaging cohort and the 28 patients in the standard cohort who achieved at least a PR to salvage therapy subsequently underwent AHSCT (Table 3). TEAM regimen was administered to 13 patients (46.4%) in the standard cohort and 36 patients (52.9%) in the imaging cohort, whereas the FEAM regimen was administered to 15 patients (53.6%) in the standard cohort and 32 patients (47.1%) in the imaging cohort (*p* = 0.56).

Patients who were resistant to second-line therapy (Table 3) were directed to further rescue treatments or experimental protocols.

### 3.4. Event-Free Survival and Outcomes

Consistent with the differences in response rates and quality between the two cohorts, the post-recurrence 2-year EFS was significantly longer in the imaging cohort compared to the standard cohort (70% [95% CI: 59–80] vs. 37.2% [95% CI: 35–55], respectively; *p* = 0.0014; Figure 1b). Cox PH analysis estimated an HR of 0.42 (95% CI: 0.25–0.72; *p* = 0.0015), indicating a 58% reduction in the hazard of relapse for patients in the imaging cohort. No differences were observed within the imaging cohort between patients monitored with PET/CT versus US/C-XR. At the last follow-up (median observation time after relapse: 30 months; median observation time after AHSCT: 2 years), 53 patients (66.2%) in the imaging cohort and 16 patients (37.2%) in the standard cohort were alive and in CR. Among those who underwent AHSCT, 15 patients in the imaging cohort and 12 patients in the standard cohort experienced disease relapse (Figure S2 and Table 2).

The difference in outcomes is unlikely to be explained by significant lead-time bias between the patient cohorts. Instead, it is primarily attributable to the high proportion of patients with clinically silent relapses in the imaging cohort and the improved response of this subgroup to salvage therapy, including AHSCT. Indeed, overall EFS was significantly longer in the imaging cohort (median EFS, 111 months [95% CI: 90–NR]) compared to the standard cohort ((median EFS, 63 months [95% CI: 50–111]); *p* = 0.023; Figure 1c), with a corresponding 46% reduction in the risk of events (HR = 0.54, 95% CI: 0.32–0.92; *p* = 0.024).

### 3.5. Impact of Symptomatic vs. Asymptomatic Relapses

**Figure 2:**
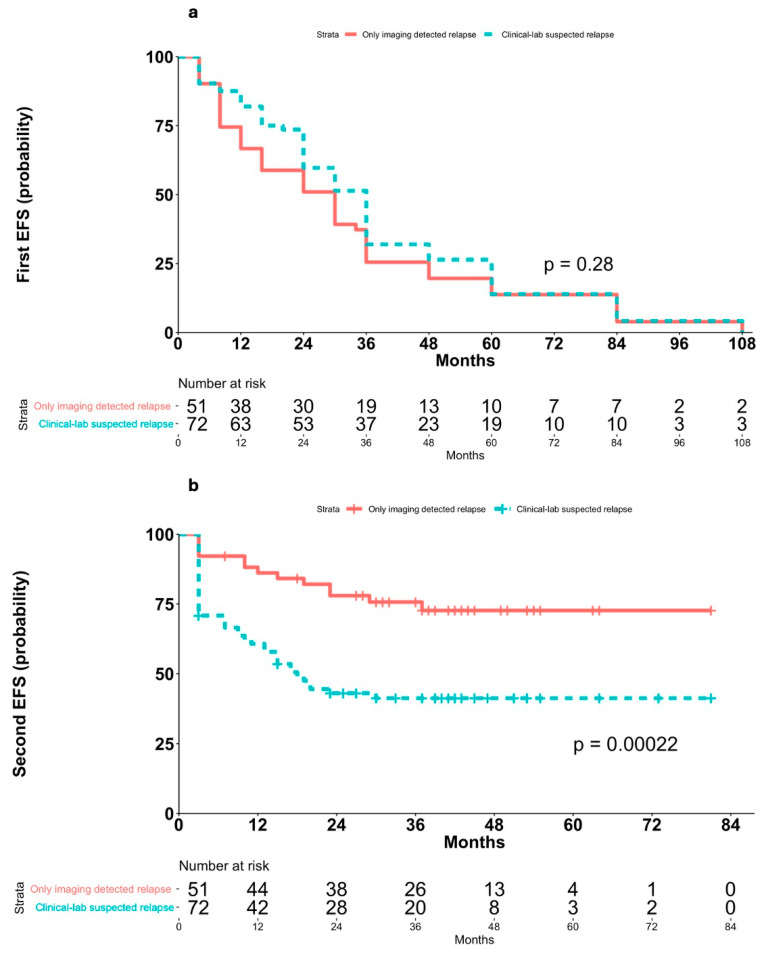
Kaplan–Meier analyses of EFS in patients who experienced asymptomatic relapses vs. those with symptomatic relapses diagnosed by the presence of clinical and/or laboratory abnormalities. (**a**) First EFS in asymptomatic relapsed patients and symptomatic patients after first complete remission; (**b**) second EFS in asymptomatic relapsed patients and symptomatic patients.

**Figure 3:**
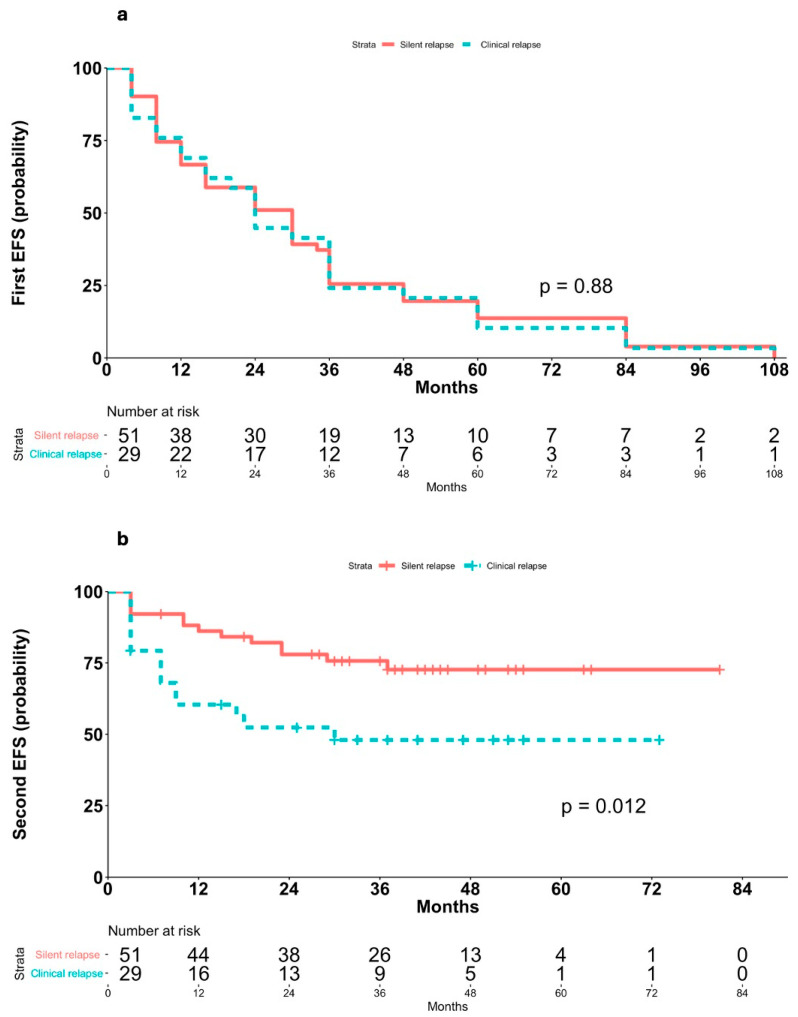
Kaplan–Meier analyses of EFS in patients who relapsed after imaging-based follow-up stratified according to clinical evident and clinical silent relapse. (**a**) First EFS according clinical evident and clinical silent relapse within the imaging cohort. (**b**) Second EFS according to clinical evident and clinical silent relapse within the imaging cohort.

Second EFS was significantly longer in patients with asymptomatic relapses (median not reached) than in those with symptomatic relapses (median 18 months, 95% CI 13–NA; *p* = 0.0002; Figure 2b), and Cox PH analysis showed a markedly increased risk of post-recurrence events in patients with clinical or laboratory abnormalities at relapse (HR 3.08, 95% CI 1.65–5.76, *p* < 0.001). This finding was confirmed within the imaging cohort, patients with clinically silent relapses had significantly longer second EFS (median not reached) compared to those with clinically evident relapses (median 30 months, 95% CI 9–NA; *p* = 0.012; Figure 3b), and Cox PH analysis confirmed a markedly increased risk in patients with clinically evident relapses (HR 2.57, 95% CI 1.20–5.49, *p* = 0.015).

This hypothesis is further supported by the finding that no significant differences were observed in first EFS between patients with asymptomatic relapses (median 36 months, 95% CI 24–36) and those with symptomatic relapses (median 30 months, 95% CI 16–36) in the overall study population (log-rank *p* = 0.28; Figure 2a; Cox HR 1.17, 95% CI 0.59–1.22, *p* = 0.4) nor between clinically silent (median 30 months, 95% CI 16–36) and clinically evident relapses (median 24 months, 95% CI 16–36) within the imaging cohort (*p* = 0.88; Figure 3a; Cox HR 1.04, 95% CI 0.66–1.64, *p* = 0.87).

### 3.6. Outcomes After PSM Sub-Analysis

A PSM analysis was performed to minimize baseline differences and better isolate the effect of follow-up strategy on survival outcomes. After matching, the cohort included 86 patients equally divided between the imaging and standard follow-up cohorts. Covariates such as age at diagnosis, histological type, stage at diagnosis, and IPS score were well balanced between groups. No significant differences in baseline characteristics remained after matching, as confirmed by standardized mean differences below 0.1 (Table S2).

**Figure 4:**
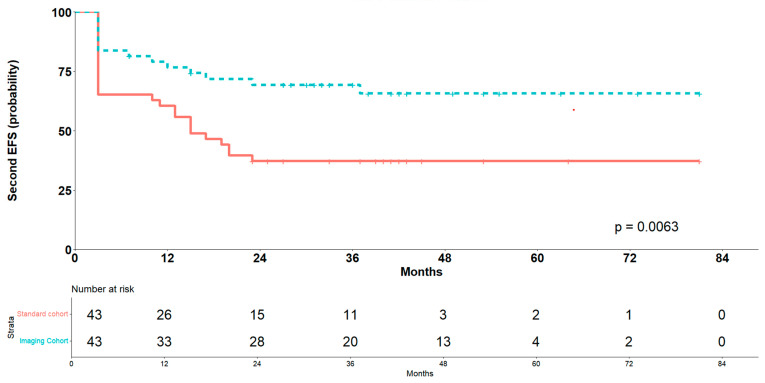
Kaplan–Meier analyses of second EFS according to method of diagnosis recurrence after propensity score matching.

Survival analysis using a Cox proportional hazards model with cluster-robust standard errors to account for intra-pair correlation demonstrated a statistically significant association between follow-up strategy and event risk. Specifically, the imaging follow-up group exhibited a 58% reduction in hazard compared to the standard follow-up group (hazard ratio 0.42; 95% CI: 0.22–0.79; *p* = 0.007). The model showed moderate discriminative ability, with a concordance index of 0.62. Kaplan–Meier survival curves after PSM confirmed this advantage for the imaging cohort (Figure 4).

These findings suggest that, in a propensity-score-matched population balanced for key prognostic factors, imaging follow-up is associated with a significant survival benefit compared to standard follow-up.

### 3.7. Prognostic Factors for Event-Free Survival

**Figure 5:**
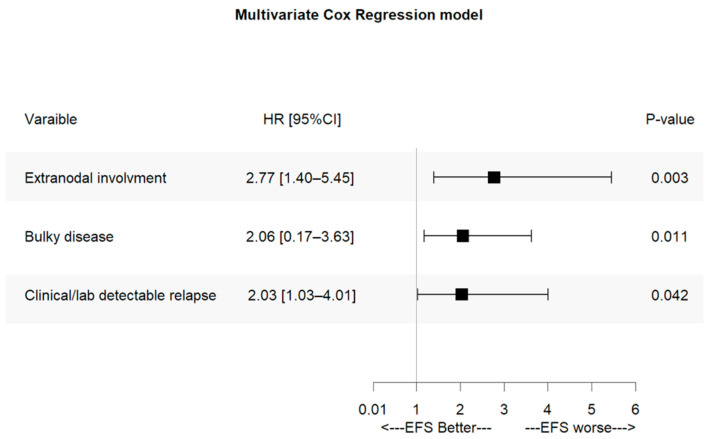
Multivariate analysis of EFS. HR, hazard ratio; 95% CI, 95% confidence interval; *p* significant as *p*-value.

Finally, we investigated relapse features predicting outcomes in the entire patient population. Several variables—including Ann Arbor stage, presence of B symptoms, bulky disease, extranodal involvement, and clinical and/or laboratory indicators of relapse (e.g., patient-reported symptoms, abnormal blood tests, and/or physical examination findings)—were associated with shorter event-free survival (EFS) in univariate analyses (Table 4). However, multivariate Cox proportional hazards modeling identified bulky disease, extranodal involvement, and symptomatic relapse as the only independent prognostic factors significantly associated with shorter EFS (Figure 5).

## 4. Discussion

We conducted a cohort study on unfavorable-risk HL patients comparing outcomes of 43 consecutive cases who relapsed under standard clinical surveillance at SMLM Hospital in Naples (Italy), with 80 relapsed cases monitored at the University Hospital Federico II of Naples using routine imaging surveillance during the same 10-year period. The latter group corresponds to the relapsed population from our previously published randomized trial comparing two post-remission imaging surveillance strategies in high-risk HL [9] (Figure S2). These two imaging follow-up strategies relied on either scheduled PET/CT scans or a combination of modern Doppler US (assessing lymph node morphology and angioarchitecture) and C-XR for mediastinal evaluation. In the referenced trial, this imaging-based approach demonstrated diagnostic accuracy comparable to PET/CT and, importantly, both methods detected subclinical relapses in approximately 60% of unfavorable-risk HL patients [9]. Therefore, the 80 relapsed patients from that trial were pooled into a single cohort to assess whether imaging-based surveillance could improve outcomes in unfavorable-risk HL.

The primary endpoint of this cohort study was second EFS, evaluated after at least two years of follow-up following AHSCT [9]. Given the growing availability of effective treatments for relapsed or refractory HL, overall survival is no longer a reliable measure of prognosis.

We acknowledge that modern frontline regimens for advanced HL, including novel agents, have improved progression-free survival, particularly in younger patients with favorable prognostic features [26,27]. However, treating cHL remains a balance to maximize cure rates while minimizing short-term and long-term toxicity. Nonetheless, relapses continue to occur, and early detection remains clinically relevant, as it can guide timely initiation of salvage therapy and influence long-term outcomes [22,23]. Despite the introduction of new lines of therapy, the prognosis of patients who experience relapse in Hodgkin lymphoma remains poor. Although overall survival has improved with the availability of agents such as brentuximab vedotin and PD-1 inhibitors [28,29], outcomes for patients with primary refractory disease or advanced-stage relapse are still inferior compared to those with therapy-sensitive disease. Our study provides real-world evidence from a large, unselected cohort, complementing clinical trial data and reflecting settings where traditional chemotherapy is still widely used. The potential benefit of imaging-based surveillance may be particularly pronounced in patients at higher risk of relapse, including older patients, those with comorbidities, or those treated outside clinical trials.

Despite this, the value of imaging surveillance for HL relapse after induction therapy remains controversial, partly due to cost concerns. Most studies have been retrospective and relied on conventional radiography or CT, which have low sensitivity for detecting relapse [4,6,8,30,31]. Even with more sensitive PET scans, findings on cost-effectiveness are conflicting [3,5,7]. Furthermore, previous studies included all HL patients regardless of disease stage, though relapses in limited-stage HL are rare (<10%) due to effective multiagent chemotherapy, making routine monitoring less justifiable in this subgroup.

Most studies on HL monitoring have been retrospective. In this study, we focused on HL patients classified as unfavorable risk at diagnosis, with a 5-year relapse risk of 20–35%, matching 26.6% and 27.5% in our imaging and standard cohorts, respectively. Notably, 51 of 80 relapses in the imaging cohort—26 detected by US/C-XR and 25 by PET/CT—were subclinical, identified without symptoms, abnormal labs, or physical findings, involving deep lymph nodes or organs. Consistent with evidence that timely salvage therapy improves long-term survival (25–90% depending on risk factors) [17,32,33,34], the imaging cohort showed significantly better outcomes: median EFS not reached vs. 15 months, CR rates pre-AHSCT of 68.8% vs. 41.9%, and end-of-study CR of 77.9% vs. 57.1%.

These results align with more limited disease at relapse in the imaging cohort (≤IIA, 57.5% vs. 32.6%), correlating with longer post-recurrence EFS. Among patients with silent relapses, stages ≤ IIA were frequent (72.5%), bulky disease and extranodal involvement were rare (13.7% and 3.9%), and outcomes were excellent, with median EFS not reached and a 2-year CR rate of 78%. Thus, clinically silent relapse is an independent positive prognostic factor for post-recurrence EFS, alongside extranodal involvement and bulky disease at relapse.

A detailed comparison between PET/CT and US/C-XR scans was presented in our previous manuscript [9]. Briefly, while PET/CT has high sensitivity for detecting suspected HL relapse, with a negative predictive value (NPV) typically between 99 and 100% [35,36], its main limitations remain its low specificity and positive predictive value (PPV) due to a high rate of false positives. Notably, combining PET with CT did not significantly reduce this issue. In our current study’s imaging cohort, these limitations were confirmed [9]. PET/CT achieved a sensitivity and NPV of 100%, but specificity and PPV were only 86.3% and 72.7%, respectively. In contrast, the combination of US with power Doppler assessment of lymph node vascularization and C-XR for mediastinal evaluation demonstrated a comparable sensitivity of 97.5%, which was not statistically different from PET/CT. Importantly, the US+C-XR approach resulted in only 4 false positives, compared to 15 in the PET/CT group.

A focused analysis of these false positives highlighted the advantage of US+C-XR in mediastinal assessment. Specifically, 11 of the 15 PET/CT false positives were due to benign lesions with glucose uptake, resulting in a mediastinal PPV of just 64.5%. These findings underscore the superior specificity and lower false positive rate of the US+C-XR surveillance approach.

The introduction of new-generation ultrasonographic devices, combining tissue harmonic imaging (which integrates signals from multiple angles) with power Doppler (which detects neoangiogenesis in lymph node tissue [9,10]), has significantly enhanced image resolution. This allows for improved detection of both non-palpable superficial and deep-seated lymphadenopathy compared to traditional grayscale ultrasound [9,10]. Although power Doppler is widely available and can be performed by operators with basic ultrasound experience, the comprehensive lymph node assessment conducted in this study was performed by a single highly experienced operator. Reproducing this systematic and detailed evaluation in routine practice may be challenging and would likely require dedicated training. C-XR also provides valuable information for assessing mediastinal disease [9]. Therefore, combining modern US with C-XR offers a safe, accurate, and cost-effective first-line imaging strategy for monitoring lymph node involvement during follow-up in high-risk HL patients.

Additional arguments against the routine use of PET or PET/CT in surveillance include (*i*) the significant exposure to ionizing radiation—already substantial from prior chemo-radiation therapy. In fact, the radiation dose required to detect a single relapse with PET/CT is estimated to be 44 times higher than C-XR, which is particularly concerning given the young age at onset and high cure rates of HL. This raises the risk of radiation-induced secondary malignancies from repeated imaging. (*ii*) The substantially higher cost, with the detection of one relapse via PET/CT being approximately 10 times more expensive for the national healthcare system.

This study compares two well-balanced cohorts of high-risk HL patients, with follow-up strategy primarily determined by institutional policy. Within the imaging cohort at Federico II University of Naples, assignment to PET/CT versus US+C-XR was performed as part of a randomized trial, ensuring group balance and reducing selection bias. Nevertheless, the retrospective design carries inherent risks of selection bias and unmeasured confounders, and treatment at different institutions may introduce center-specific variability. To address potential baseline confounding, we performed PSM sub-analysis. In the matched cohort, imaging-based follow-up remained significantly associated with improved survival outcomes, reinforcing the robustness of our primary findings while acknowledging that residual confounding inherent to the retrospective design cannot be entirely excluded. We also acknowledge that both participating institutions are located within the same geographic region, which may limit the generalizability of our findings to populations with different demographic, clinical, or treatment characteristics

However, further limitations should be acknowledged. Although second EFS was significantly improved in the imaging-based group, overall survival data were not mature enough to determine whether early relapse detection translates into a long-term survival advantage. We chose not to present OS as an endpoint because the heterogeneity of therapies administered after salvage treatment could confound its interpretation between study groups. Longer-term follow-up and prospective studies, including cohorts from diverse geographic regions and healthcare settings, are required to confirm the sustained survival benefit associated with imaging-based follow-up and to validate the broader applicability of our findings. While the imaging modalities were compared for diagnostic accuracy and clinical outcomes, no formal cost-effectiveness analysis was performed; therefore, conclusions regarding economic impact are indirect and may vary depending on healthcare system structures.

## 5. Conclusions

Our study shows that, in high-risk HL patients, imaging-based follow-up after remission is superior to standard clinical surveillance in terms of earlier detection of relapse. This earlier identification enables treatment at a less advanced stage, resulting in significantly better outcomes following salvage therapy. Given that most relapses occur within the first 36 months, we propose a follow-up strategy combining US and C-XR with clinical evaluation and blood tests during the first three years after first complete remission. Moreover, time to relapse was similar between symptomatic and asymptomatic patients, suggesting that the improved post-recurrence EFS in those with imaging-detected relapse is not due to lead-time bias but, rather, to limited disease burden with more effective salvage treatment.

## Figures and Tables

**Table 1:** Clinical features of the two cohorts of patients at initial diagnosis of Hodgkin lymphoma.

	Standard Cohort (*n* = 43)	Imaging Cohort (*n* = 80)	*p*-Value
**Sex**			
Female	22 (51.2)	44 (55)	0.78
Male	21 (48.8)	36 (45)	
**Age, years**			
Median, (range)	30 (19–68)	31 (20–66)	0.96
Elderly patients (>60 years old)	2 (4.7)	5 (6.2)	0.13
**Histological type**			
Nodular sclerosis	27 (62.8)	50 (62.5)	0.92
Mixed cellularity	12 (27.9)	22 (29.7)	
Nodular lymphocyte predominant	2 (4.7)	2 (2.5)	
Lymphocyte-rich	1 (2.3)	4 (5)	
Lymphocyte-depleted	1 (2.3)	2 (2.5)	
**Ann Arbor stage**			
IIB	8 (18.6)	18 (22.5)	0.40
IIIA	11 (25.6)	18 (22.5)	
IIIB	12 (27.9)	28 (35)	
IVA	4 (9.3)	10 (12.5)	
IVB	8 (18.6)	6 (7.5)	
**Risk factors**			
Involvement of ≥ 3 nodal areas	37 (86)	70 (87.5)	0.82
Bulky disease ^†^	26 (60.5)	54 (67.5)	0.43
High erythrocyte sedimentation rate ^‡^	27 (62.8)	54 (67.5)	0.59
Extranodal involvement (in stages < IV)	14 (32.6)	31 (38.8)	0.46
**International Prognostic Score (grouped)**			
0–1	13 (30.2%)	26 (32.5)	0.96
2–3	17 (39.5%)	30 (37.5)	
4–7	13 (30.2%)	24 (30)	

^†^ Defined as single lymph node or conglomerate nodal mass of ≥5 cm long axis at CT. ^‡^ Defined as ≥50 mm/h without fever, sweating, and weight loss; ≥30 mm/h with fever, sweating and weight loss.

**Table 2:** Clinical features of the two cohorts of patients at the relapse restaging.

	Standard Cohort (*n* = 43)	Imaging Cohort (*n* = 80)	*p*-Value	Imaging Cohort		*p*-Value
				Clinical Relapses (*n* = 29)	Clinically Silent Relapses (*n* = 51)	
**Median time to relapse, months (range)**	36 (4–108)	24 (4–108)	0.012	34 (4–84)	24 (4–108)	0.02
**Ann Arbor stage**						
≤IIA	14 (32.6)	46 (57.5)	0.008	9 (31.0)	37 (72.5)	0.0003
≥IIB	29 (67.4)	34 (42.5)		20 (69.0)	14 (27.5)	
**Nodal regions involved at restaging as detected by FDG-PET scans after relapse**						
Superficial	24 (55.8)	35 (43.8)	0.201	15 (51.7)	20 (39.2)	0.278
Mediastinal compartment	25 (58.1)	35 (43.8)	0.128	18 (62.1)	17 (33.3)	0.013
Abdominal compartment	26 (60.5)	45 (56.3)	0.652	19 (65.5)	26 (51.0)	0.207
**Unfavorable prognostic factors**						
Involvement of ≥3 nodal areas	20 (46.5)	33 (41.3)	0.574	14 (48.3)	19 (37.3)	0.336
Bulky disease ^†^	25 (58.1)	19 (23.8)	0.0003	12 (41.4)	7 (13.7)	0.005
Extranodal involvement (in stages < IV)	9 (20.9)	7 (8.8)	0.055	5 (17.2)	2 (3.9)	0.043
**Residual mass sites involved at relapse**	10 (23.3)	16 (20.0)	0.673	6 (20.7)	10 (19.6)	0.907
**Involvement of extranodal regions at relapse**	14 (32.6)	15 (18.8)	0.085	8 (27.6)	7 (13.7)	0.123

Note: Unless otherwise indicated, data are number of patients, with percentage in parentheses. ^†^ Defined as single lymph node or conglomerate nodal mass of ≥5 cm long axis at CT.

**Table 3:** Outcome of patients of the two cohorts after salvage therapy and at last follow-up.

	Salvage Therapy			Last Follow-Up After AHSCT	
	Overall Response Rate	Complete Remission	*n*. AHSCT	Complete Remission	Relapse
Standard cohort (*n* = 43)	28 (65.1)	18 (41.9)	28 (65.1)	16 (57.1)	12 (42.9)
Imaging cohort (*n* = 80)	68 (85)	55 (68.8)	68 (26.3)	53 (77.9)	15 (22.1)
Clinical relapses (*n* = 29)	21 (72.4)	12 (41.4)	21 (72.4)	13 (61.9)	8 (38.1)
Clinically silent relapses (*n* = 51)	47 (92.2)	43 (84.3)	47 (92.2)	40 (85.1)	7 (14.9)

**Table 4:** Univariate hazard ratios (HR) for EFS after relapses in the entire study population.

Relapse Characteristics	Univariate Analysis	
	HR (95% CI)	*p*-Value
**Relapse detection method**		
Clinical/lab positive vs. imaging only	3.07 (1.64–5.75)	0.00044
Standard vs. imaging follow-up	0.42 (0.25–0.75)	0.0015
**Ann Arbor stage at relapse**		
≥IIB vs. ≤IIA	2.19 (1.26–3.81)	0.006
B-symptoms vs. no B-symptoms	1.80 (1.05–30.8)	0.031
**Unfavorable prognostic factors**		
Involvement of ≥3 vs. <3 nodal areas	1.16 (0.68–1.99)	0.575
Bulky disease ^†^ vs. no bulky disease	2.59 (1.51–4.42)	0.0005
Extranodal involvement vs. no extranodal disease	3.65 (1.90–7.08)	0.0006
ESR > 50 mm/s vs. ESR ≤ 50 mm/s	2.28 (1.31–3.97)	0.003
IPS 4–7 vs. 2–3 vs. 0–1	1.29 (0.92–1.81	0.13

^†^ Defined as single lymph node or conglomerate nodal mass of ≥5 cm long axis at CT.

## Data Availability

The data presented in this study are available on request from the corresponding author due to privacy restrictions.

## Supplementary Materials

The following supporting information can be downloaded at: https://www.mdpi.com/article/10.3390/cancers17193242/s1, Figure S1: Timeline of post-remission follow-up; Figure S2: Study flow chart. The figure shows the management diagram of the whole group of patients followed since diagnosis in the two institution; Table S1: Clinical features at diagnosis of the HL patients followed either at the Hospital of the University of Naples Federico II with a systematic imaging-based surveillance or at the Santa Maria di Loreto Mare Hospital of Naples with the standard clinical-based surveillance; Table S2: Covariate balance before and after propensity score matching between imaging and standard follow-up cohorts.

## Abbreviations

The following abbreviations are used in this manuscript:

CR	Complete response
HL	Hodgkin lymphoma
AHSCT	Autologous hematopoietic stem-cell transplant
ABVD	Adriamycin, bleomycin, vinblastine, and dacarbazine
US	Ultrasonography
FDG	Fluorodeoxyglucose computed tomography (CT) scans
PET	Positron emission tomography
CT	Computed tomography
ESR	Erythrocyte sedimentation rate
PR	Partial response
EFS	Event-free survival
95% CI	95% confidence interval
HR	Hazard ratio
PH	Proportional hazards
PMS	Propensity score matched
