# Supplementary material for: Routine Imaging Surveillance After Frontline ABVD Improves Outcome in High-Risk Hodgkin Lymphoma
Source: Cancers (Basel). 2025 Oct 6;17(19):3242. doi: 10.3390/cancers17193242 (PMC12524035; doi:10.3390/cancers17193242)
Supplement: Supplementary file 1 [file cancers-17-03242-s001.zip › cancers-3890622-supplementary.pdf]

Supplemental method

Follow-up imaging procedures in the US/chest radiography (US/C-XR) group comprised:

1) Ultrasonography (US): systematic evaluation of superficial, anterosuperior mediastinal, abdominal, and pelvic lymph nodes. Mediastinal assessment included clavicular, supra-aortic, and prevascular nodal regions (anterosuperior compartment), as well as subaortic, para-aortic, hilar, subcarinal, and paraesophageal nodal regions (deep compartment). Abdominal and pelvic assessment included mesenteric, hepatic hilum, para-aortic/paracaval, and iliac nodal regions, as well as liver and spleen.
2) Chest radiography (C-XR): frontal and lateral chest radiographs were obtained to evaluate mediastinal compartments. Conventional radiographic criteria previously described [9] were applied to define positive or negative findings.

All US examinations were performed by a hematologist (M.P.) with >10 years of expertise in diagnostic US for superficial and deep-seated lymph nodes, using an iU22 scanner (Philips Healthcare, Bothell, WA) equipped with tissue harmonic compound technology (SonoCT; Philips), power Doppler, and 5–2-MHz (C5–1 curvilinear) and 9–3-MHz (L9–3 linear) broadband probes. Each complete examination required approximately 40 minutes (range, 30–60 minutes).

Diagnostic criteria: Lymph nodes were considered positive for HL recurrence if ≥3 of the following features were present: (a) long axis ≥1.5 cm, (b) round shape, (c) absence of hilum, (d) hypoechoic parenchyma, (e) hypervascularization with intranodal arterial vessels and resistive index ≥0.6 [9].

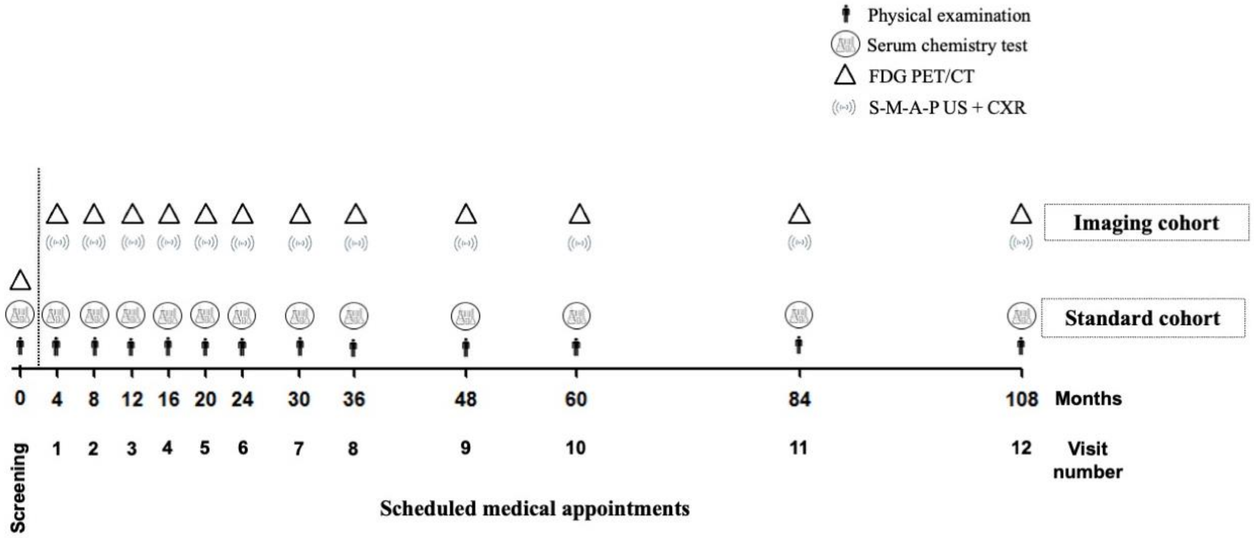

Figure S1. Timeline of post-remission follow-up.

Scheduled surveillance visits occurred at months 4, 8, 12, 16, 20, 24, 30, 36, 48, 60, 84, and 108 after completion of treatment. Patients in the imaging cohort underwent systematic imaging assessments using PET/CT or ultrasonography combined with chest radiography (US/C-XR), in addition to clinical evaluation and laboratory testing. Patients in the standard follow-up cohort were monitored through clinical evaluation and laboratory testing, with imaging performed only when there was clinical suspicion of relapse.

Supplemental data

Table S1 summarizes the baseline patient-, disease-, and therapy-related characteristics of 456 patients with high-risk HL at diagnosis. Approximately 75% of patients were stage III or IV, while the remainder in each group had stage IIB with one or more risk factors. After scheduled ABVD, radiation therapy was administered to 59 of 98 (60.2%) patients in the standard-cohort and 131 of 211 (62.1%) patients in the imaging-cohort who presented with bulky disease. Overall, 30% of patients showed PET-negative residual masses following completion of first-line treatment. After remission, patients were followed with either systematic imaging-based surveillance (n = 300) or classical clinical surveillance (n = 156) (Figure S2).

Among 315 patients with high-risk HL at diagnosis treated at the University of Naples Federico II who achieved CR after first-line therapy, 15 (4.8%) were excluded from follow-up: seven declined participation, and eight were excluded due to comorbidities impairing follow-up procedures (two had diabetes mellitus unresponsive to hypoglycemic treatment, and six had obesity). At the SMLM center, among 160 patients with high-risk HL achieving CR after first-line therapy, four (2.6%) declined participation. There was no significant difference in exclusion rates between the two follow-up groups (*P*= 0.26).

Table S1. Clinical features at diagnosis of the HL patients followed either at the Hospital of the University of Naples Federico II with a systematic imaging-based surveillance or at the Santa Maria di Loreto Mare Hospital of Naples with the standard clinical-based surveillance.

	Standard cohort (n= 156)	Imaging cohort (n= 300)	*P* value
**Sex**			
Female	65 (41.7)	119 (39.7)	0.68
Male	91 (58.3)	181 (60.3)	
**Age, years**			
Median, (range)	30 (19-73)	29 (18-70)	0.55
Elderly patients (> 60 years old)	19 (12.2)	38 (12.6)	0.09
**Histological type**			
Nodular sclerosis	98 (62.8)	187 (62.3)	
Mixed cellularity	49 (31.4)	89 (29.7)	
Nodular lymphocyte predominant	4 (2.6)	10 (3.3)	0.87
Lymphocyte-rich	2 (1.3)	8 (2.7)	
Lymphocyte-depleted	3 (1.9)	6 (2.0)	
**Ann Arbor stage**			
IIB	34 (22.1)	55 (18.3)	
IIIA	32 (20.5)	75 (25.0)	
IIIB	37 (23.7)	85 (28.3)	0.63
IVA	13 (8.3)	20 (6.7)	
IVB	40 (25.6)	65 (23.3)	
**Risk factors**			
Involvement of ≥ 3 nodal areas	136 (87.2)	262 (87.3)	0.96
Bulky disease†	98 (62.8)	211 (70.3)	0.10
High erythrocyte sedimentation rate‡	101 (65.2)	208 (69.3)	0.32
Extranodal involvement (in stages < IV)	53 (33.9)	122 (40.7)	0.16
**International Prognostic Score (grouped)**			
0-1	43 (27.6%)	91 (30.3)	
2-3	72 (46.2%)	121 (40.3)	0.49
4-7	41 (26.3%)	88 (29.3)	
**First-line treatment**			
Induction chemotherapy, six courses	156 (100)	300 (100)	
Bulky site irradiation after chemotherapy	59 (37.8)	131 (43.7)	0.23
**Post-treatment assessment**			
No focal FDG uptake at PET	156 (100)	300 (100)	0.21
Residual masses at CT§	43 (27.6)	100 (33.3)	

Note: Unless otherwise indicated, data are number of patients, with percentage in parentheses.

† Defined as single lymph node or conglomerate nodal mass of ≥ 5 cm long axis at CT.

‡ Defined as ≥ 50 mm/h without fever, sweating, and weight loss; ≥ 30 mm/h with fever, sweating and weight loss.

§ Defined as nodes ≥ 1.3 cm long axis at CT after scheduled treatment

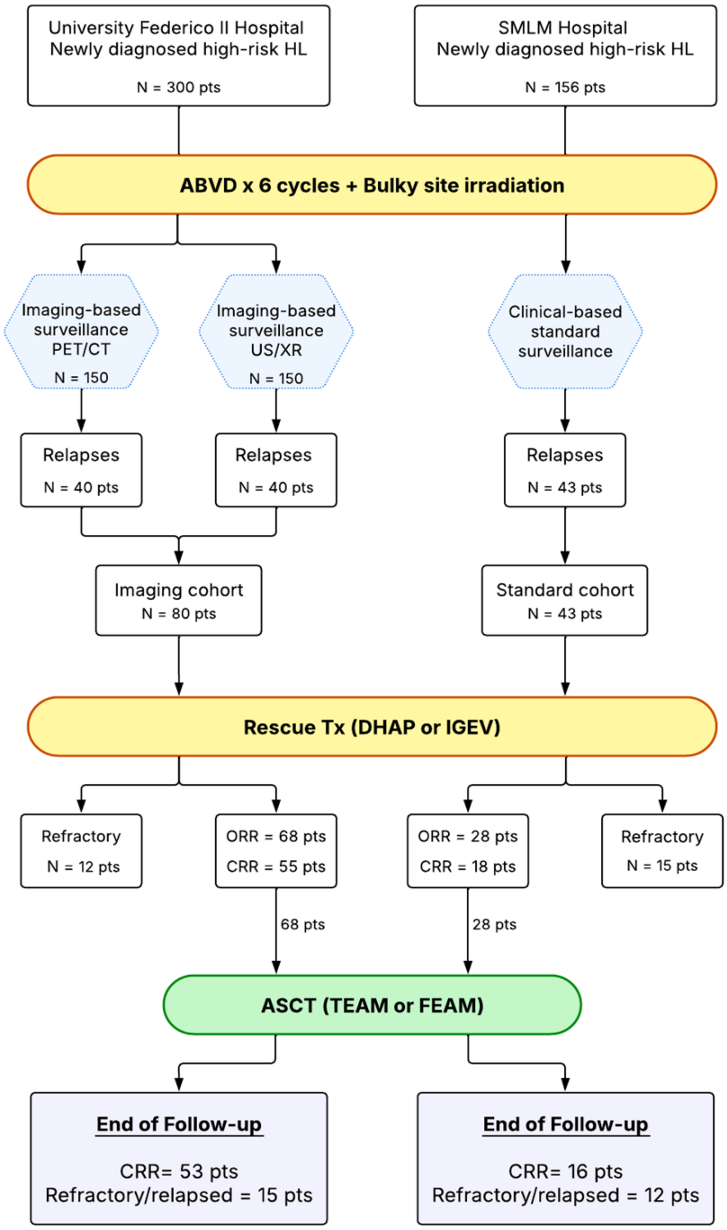

Figure S2. Study flow chart. The figure shows the management diagram of the whole group of patients followed since diagnosis in the two institution.

Table S2. Covariate balance before and after propensity score matching between imaging and standard follow-up cohorts

Covariate	Imaging Cohort Mean (Before Matching, n= 80)	Standard Cohort Mean (Before Matching, n= 43)	Std. Mean Diff. Before Matching	Imaging Cohort Mean (After Matching, n= 43)	Standard Cohort Mean (After Matching, n= 43)	Std. Mean Diff. After Matching
**Age at diagnosis**	33.69	32.70	0.091	31.98	32.70	-0.066
**Histological type**	0.575	0.535	0.042	0.488	0.535	-0.049
**Stage at diagnosis**	1.60	1.84	-0.200	1.74	1.84	-0.078
**IPS score**	0.975	1.00	-0.031	1.00	1.00	0.000[i]

[i] **Note:** Std. Mean Diff. (standardized mean difference); values < 0.1 indicate good covariate balance.
